# Single breath-hold Vd(m) calculation as good as multi breath-hold technique in Equilibrium Contrast CMR

**DOI:** 10.1186/1532-429X-14-S1-P262

**Published:** 2012-02-01

**Authors:** Daniel Sado, Stefan K Piechnik, Matthew D Robson, Viviana Maestrini, Andrew Flett, Steven K White, Sanjay M Banypersad, James Moon

**Affiliations:** 1 Imaging Centre, The Heart Hospital, London, UK; 2 Cardiovascular Medicine, Centre for Clinical Magnetic Resonace Research, Oxford, UK

## Summary

In this study we have compared the multi flip angle (MFA) FLASH T1 mapping technique to shortened look locker inversion recovery (ShMOLLI) for assessment of pre and post contrast T1 for equilibium contrast CMR (EQ-CMR) and also potential pseudoequilibrium post bolus. Good agreement was found in the volume of distribution, Vd(m), between both techniques and at equilibirum.

## Background

Interstitial fibrosis can be measured by the assessment of Vd(m) and has been shown to be accurate comparing EQ-CMR against histology. However, this technique utilized an MFA FLASH T1 mapping technique, requiring 7 breath-holds per slice to measure T1. Newer techniques such as ShMOLLI promise faster measurement.

## Methods

We compared Vd(m) calculated from T1 pre and post contrast administration using T1 mapping with multi-breath hold MFA FLASH and single breath-hold ShMOLLI in a cohort of normal volunteers on a 1.5T Siemens Avanto scanner. We also compared ShMOLLI Vd(m) at equilibrium with that obtained 15 minutes after gadolinium bolus, a potential pseudoequilibrium [1].

## Results

**Figure 1:**
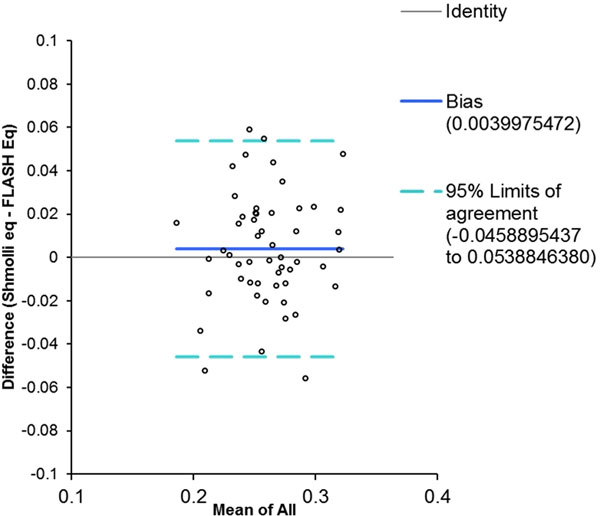
Bland Altman plot: ShMOLLI Vs FLASH Vd(m)

**Figure 2:**
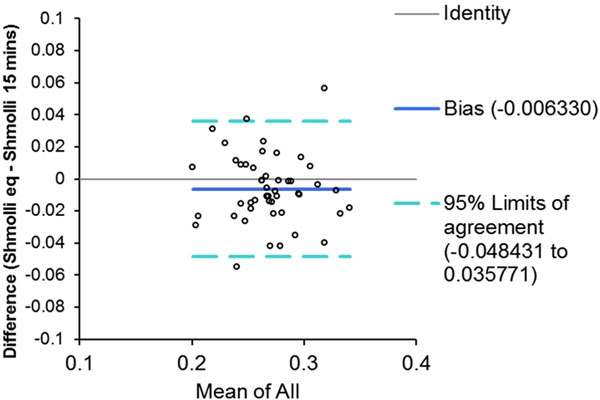
Bland Altman plot: ShMOLLI equilibrium Vd(m) Vs pseudoequilibrium

54 subjects (median age 49, range 24-81, 26 males) were recruited. We noted poor agreement and correlation in the pre contrast T1s in both blood and myocardium. There was strong correlation, but poor agreement in post contrast T1 measurements. However, mean Vd(m) at equilibrium was 0.258±0.032 using MFA FLASH and 0.263±0.034 with ShMOLLI, with good agreement on Bland Altman analysis (Fig 1), suggesting that the Vd(m) calculation can compensate for the variability in pre contrast T1 measurements. There was also good agreement between the Vd(m) at equilibrium and at potential pseudoequilibrium (Fig 2).

## Conclusions

The good agreement between MFA FLASH and ShMOLLI is the first evidence for a role of the latter in providing accurate Vd(m) assessment using the EQ-CMR technique. This would be advantageous as it allows whole heart mapping in 3 short breath holds. The poor pre contrast T1 agreement may reflect the limitations of MFA FLASH based mapping in the assessment of tissues with long T1. The good agreement between equilibrium and potential pseudoequilibrium Vd(m) builds on previous work showing that contrast infusion is not necessary for Vd(m) assessment in healthy volunteers, however further work is required to investigate whether or not this is the case in cardiac disease.

## Funding

1) British Heart Foundation.

2) GlaxoSmithKline.

## References

[B1] Schelbert J Cardiovasc Magn Reson 2011 13 16 10.1186/1532-429X-13-16 PMC3059279 21375743

